# La-based perovskites for capacity enhancement of Li–O_2_ batteries

**DOI:** 10.3389/fchem.2023.1264593

**Published:** 2023-09-01

**Authors:** Bing-Ze Hsu, Jun-Kai Lai, Yi-Hsuan Lee

**Affiliations:** Department of Mechanical Engineering, National Taipei University of Technology, Taipei, Taiwan

**Keywords:** Li-O_2_ battery, perovskite catalysts, battery capacity enhancement, carbon black, Li_2_O_2_ and Li_2_CO_3_

## Abstract

Li–O_2_ batteries are a promising technology for the upcoming energy storage requirements because of their high theoretical specific energy density of 11,680 Wh kg^−1^. Currently, the actual capacity of Li–O_2_ batteries is much lower than this theoretical value. In many studies, perovskites have been applied as catalysts to improve the air electrode reactions in Li–O_2_ batteries. The effects of structure and doping on the catalytic activity of perovskites are still unclear. La_1-x_Sr_x_CoO_3-δ_ (*x* = 0.1, 0.3, and 0.5) and La_0.9_Sr_0.1_YbO_3-δ_ mixed with carbon black (Vulcan XC500 or Super P) were used as air electrode catalysts. Electrochemical characterizations were conducted using a Swagelok-type cell. The charge–discharge capacity and cyclic voltammetry (CV) performance were investigated in this study. The La_1-x_Sr_x_CoO_3-δ_ (*x* = 0.1, 0.3, and 0.5) is a suitable cathode catalyst for Li–O_2_ batteries. In this study, the La_0.5_Sr_0.5_CoO_3-δ_/Super P cathode demonstrated the highest discharge capacity (6,032 mAh g^−1^). This excellent performance was attributed to the large reaction area and enhanced Li_2_CO_3_ generation.

## Highlights

• La-based perovskites mixed with carbon black were used as cathode catalysts in Li–O2 batteries.
• The cathode with La0.5Sr0.5CoO3-δ/Super P demonstrated the highest charge–discharge capacity.
• Production of Li2CO3 increased discharge capacity.

## 1 Introduction

Lithium-ion batteries (LIBs) are widely utilized in laptops, smartphones, power banks, renewable energy systems, and electric vehicles worldwide (Lu et al., 2013; Li et al., 2018; Dunn et al., 2021; Muruganantham et al., 2022; Zhu et al., 2022; Anirudha et al., 2023). However, the density of energy storage in LIBs is still insufficient to fulfill the increasing energy requirements for advanced electric transportation (Wei et al., 2021; Luo et al., 2022). The recently developed metal–air batteries, including Zn–oxygen, Na–oxygen, and Li–oxygen (Li–O_2_) batteries, have attracted considerable interest because of their advantages such as low cost, high flexibility, and high theoretical energy density (Hardin et al., 2013; Rahman et al., 2013; Kang et al., 2022; Li et al., 2022; Salado and Lizundia, 2022; Yang et al., 2022). In addition, in comparison with LIBs, Li–O_2_ batteries offer much higher gravimetric energy density, which could reach the theoretical value of 11,680 Wh kg^−1^ because oxygen electrodes can directly use oxygen from the surrounding environment while discharging; therefore, oxygen does not need to be stored within the battery (Girishkumar et al., 2010). However, Li–O_2_ batteries have been facing several serious challenges, including high overvoltage, poor rate capacity, and short cycle life, which are mainly caused by the sluggish dynamics of the air electrode during Li_2_O_2_ formation (2Li^+^ + 2e^−^ + O_2_ → Li_2_O_2_, oxygen reduction reaction (ORR)) and Li_2_O_2_ decomposition [Li_2_O_2_ → 2Li^+^ + 2e^−^ + O_2_, oxygen evolution reaction (OER)] (Thapa et al., 2010; Thapa and Ishihara, 2011; Pan et al., 2019; Cui et al., 2021; Yin et al., 2021; Zhan et al., 2021). Furthermore, battery performance is also affected by the components present in ambient air, resulting in significantly lower practical specific energy (Zhang et al., 2018).

Recently, Li–O_2_/CO_2_ batteries have attracted considerable attention because they capture and utilize carbon (Takechi et al., 2011; Lim et al., 2013; Yin et al., 2017; Zou et al., 2019; Chen et al., 2020; Savunthari et al., 2021; Iputera et al., 2022; Wang et al., 2023; Wu et al., 2023). In addition, during discharge, Li^+^ ions react with O_2_ and CO_2_ to produce Li_2_O_2_ and Li_2_CO_3._ These products can improve the battery capacity by 289% compared to batteries operating on 100% O_2_ (Takechi et al., 2011). Therefore, the generation of Li_2_CO_3_ is beneficial for battery capacity. At the same time, Li_2_O_2_ and Li_2_CO_3_ that precipitate on the air electrode surface during discharge are difficult to completely decompose during charge (Gallant et al., 2012). These discharge products (Li_2_O_2_ and Li_2_CO_3_) block the pores of the air electrode and thus cause performance degradation because they hinder air supply and liquid electrolyte diffusion (Liu et al., 2017; Zhao et al., 2018). Hence, it is necessary to promote Li_2_CO_3_ generation on oxygen electrodes during discharge and decomposition of discharge products during charge.

Because reactions on the air electrode significantly affect battery performance, many studies have focused on enhancing the electrocatalytic activity of the oxygen electrode. Therefore, catalyst addition to oxygen electrodes in Li–O_2_ batteries is necessary to improve the electrochemical activities of ORR during discharge and OER during charging (Thapa et al., 2010; Thapa and Ishihara, 2011; Pan et al., 2019; Yin et al., 2021). Currently, Pt-based catalysts are considered excellent catalysts for the ORR and OER (Wu and Yang, 2013; Liu et al., 2019), and IrO_2_ is known to be the best catalyst for the OER (Lee et al., 2012). Although these materials show excellent performance, their high cost limits their application in Li–O_2_ batteries. Recently, some studies have demonstrated that perovskite catalysts are beneficial for oxygen reduction during discharge and the decomposition of discharge products during charge (Xu et al., 2013; Du et al., 2014; Jin et al., 2014; Zhang et al., 2014; Ma et al., 2020; Du et al., 2021). Oxygen vacancies in the perovskite LaCoO_3_ have been reported to enhance the bifunctional catalytic activity (ORR and OER) because of the valence electron transformation of the Co ions (Du et al., 2021). In addition, the battery discharge capacity and long-term cycling stability were also remarkably increased. Zhang et al. used porous LaNiO_3_ as a catalyst for an air electrode and improved the discharge capacity from 2,545 to 3,407 mAh g^−1^ (Zhang et al., 2014). However, it is unclear how the catalysts affect the discharge products and battery capacity.

In this study, La_1-x_Sr_x_CoO_3-δ_ (*x* = 0.1, 0.3, and 0.5) and La_0.9_Sr_0.1_YbO_3-δ_ were used as catalysts for oxygen electrode to determine their effects on Li–O_2_ battery capacity. Additionally, the performances of two types of carbon black as cathode substrates were tested. Finally, the discharge products were analyzed to explain the differences in battery performance for different cathode materials. The findings of this study are expected to facilitate the development of catalysts for air electrodes in Li–O_2_ batteries.

## 2 Experimental

### 2.1 Preparation of perovskite materials

The sol–gel method was used to synthesize La_1-x_Sr_x_CoO_3-δ_ (*x* = 0.1 (L9SC), 0.3 (L7SC), and 0.5 (L5SC)) and La_0.9_Sr_0.1_YbO_3-δ_ (L9SYb) catalysts. La(NO_3_)_2_ · 6H_2_O (purity 99.9%, Alfa Aesar, United States), Sr(NO_3_)_2_ (purity 99%, Alfa Aesar, United States), Co(NO_3_)_2_ · 6H_2_O (purity 98%, Acros Organics, United States), Yb(NO_3_)_3_⋅6H_2_O (purity 99.9%, Strem Chemicals Inc., United States), citric acid (J. T. Baker, United States), and ethylenediaminetetraacetic acid (Alfa Aesar, Spain) were separately dissolved in deionized water. The precursor solution was heated under stirring at 200°C until a gel-like phase was obtained. The dry gel was completely burned at 300°C to form a powder, which was then ground and calcination in air at 1,100°C for 5 h to obtain the L9SC, L7SC, L5SC, and L9SYb powders.

### 2.2 Characterization

The phase compositions of the synthesized materials were identified by X-ray diffraction (XRD; Malvern Panalytical Empyrean, Cu Kɑ). All the as-prepared materials were carefully sieved, and the sub-25-µm fraction was used in the XRD analysis. In addition, the compositions of cathode materials were determined using XRD before and after one charge–discharge cycle.

### 2.3 Electrochemical measurements

Electrochemical characterization was conducted using a Swagelok-type cell. The cathode was formed by casting a mixture of La-based perovskites, carbon black (Vulcan XC500 or Super P), and polytetrafluoroethylene (wt. ratio of 4.25:4.25:1.5) and then pressing the mixture onto a carbon paper (GD210, CeTech Co., Ltd., Taiwan). Lithium foil was used as the anode and was separated with a porous polypropylene film (FinTech Co., Ltd. Taiwan). Electrochemical measurements were performed using gastight Swagelok-type cells, with the exception of a stainless-steel window that enabled exposure to O_2_ gas. Lithium bis(trifluoromethanesulfonyl) (1 M in tetraethylene glycol dimethyl ether) was used as the electrolyte. The charge–discharge performance was determined in the voltage range of 2.4–4.3 V at a constant current of 0.1 mA cm^−2^ in O_2_ atmosphere.

CV was performed using the same Swagelok cell at a scan rate of 0.1 mV s^−1^ in the voltage range of 2.0–4.5 V on the Princeton V3.

## 3 Results and discussion

**FIGURE 1:**
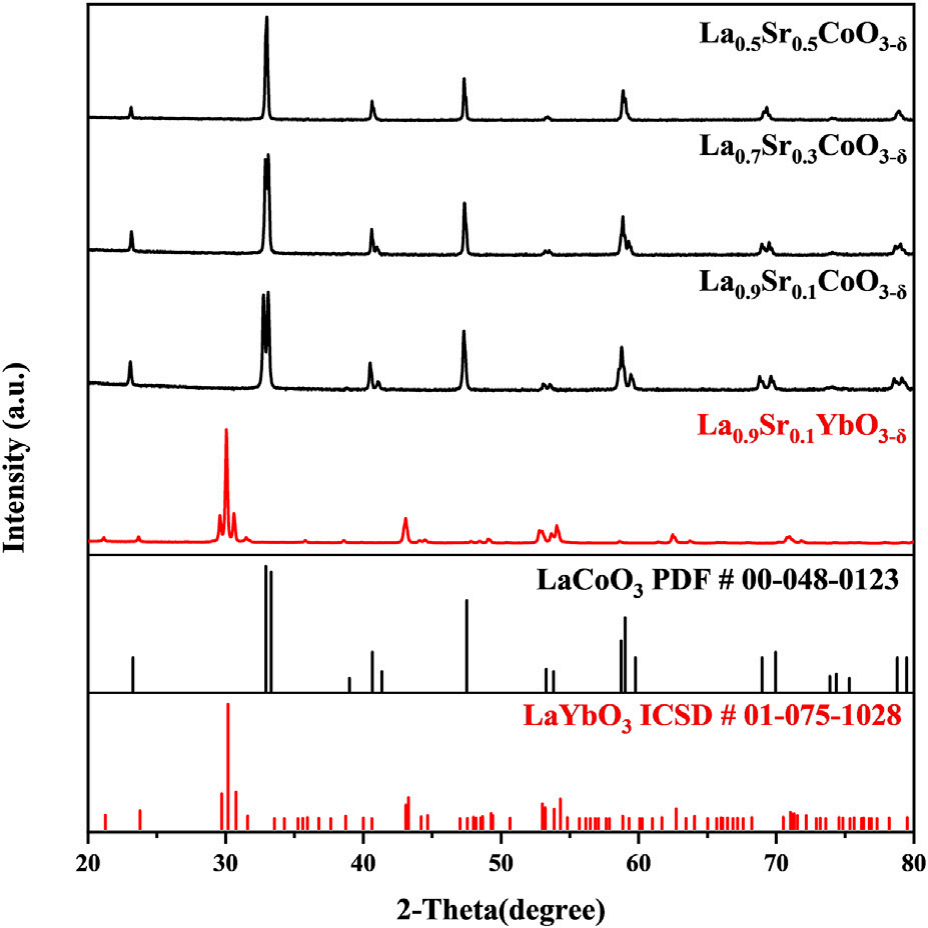
XRD patterns of the La_1-x_Sr_x_CoO_3-δ_ (*x* = 0.1, 0.3 and 0.5) and La_0.9_Sr_0.1_YbO_3-δ_.

The phase purity of L9SC, L7SC, L5SC, and L9SYb powders was verified by analyzing their crystal structures using XRD (Figure 1). The diffraction peaks of L9SC, L7SC, L5SC, and L9SYb phases match well with the corresponding reference patterns thus confirming that each as-prepared material is composed of a single major phase. In the case of La_1-x_Sr_x_CoO_3-δ_, the formation of impurity phases with increasing Sr doping is not observed, as shown in Figure 1. Therefore, the as-prepared materials have sufficiently high purities and crystallinities for use as catalysts.

**FIGURE 2:**
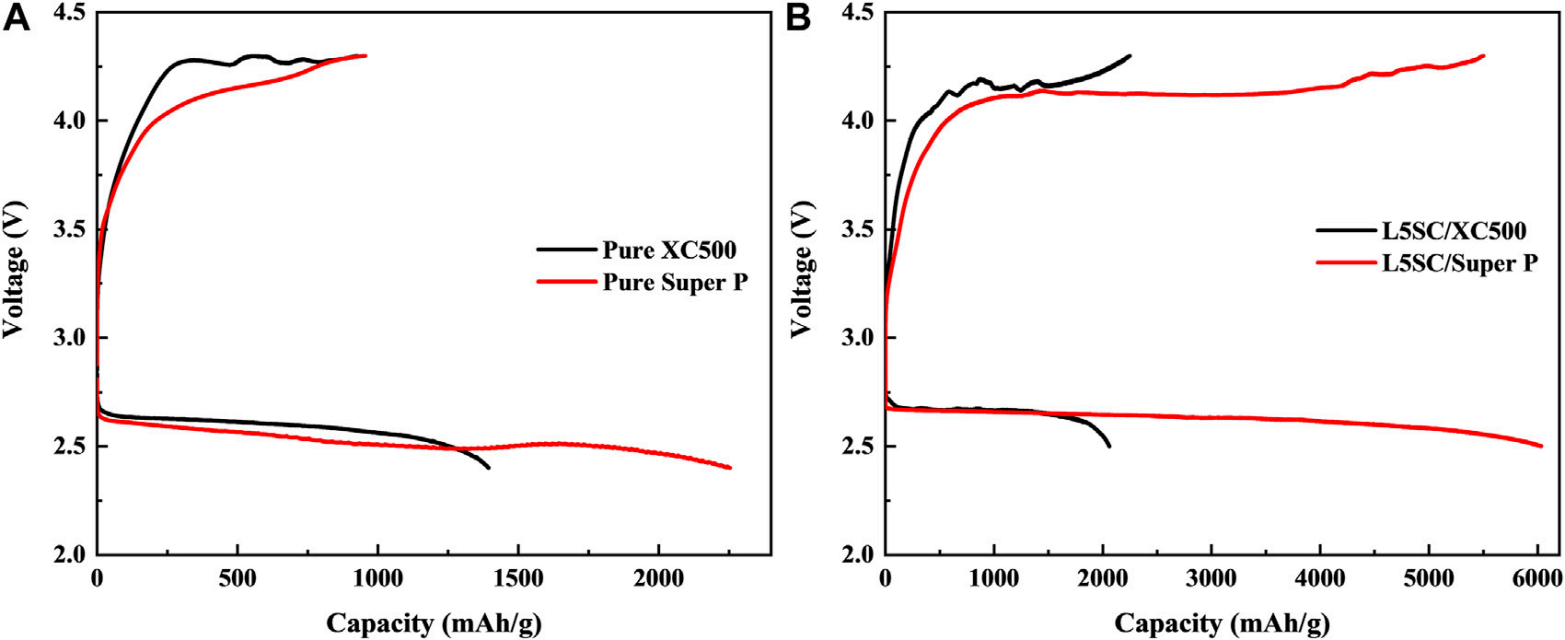
Charge–discharge curves for Li–O_2_ with different air electrodes in the voltage range of 4.3–2.4 V at a constant current of 0.1 mA cm^−2^ at room temperature. **(A)** Carbon black (Vulcan XC500 or Super P) without catalysts used as the air electrode. **(B)** Carbon black (Vulcan XC500 or Super P) with L5SC catalyst used as the air electrode.

Figure 2 shows the charge–discharge curves for Li–O_2_ in the voltage range of 4.3–2.4 V at a constant current of 0.1 mA cm^−2^ at room temperature. First, only carbon black (Vulcan XC500 and Super P) electrodes without catalysts were tested in one-cycle charge-discharge tests (Figure 2A). The charge–discharge capacity results are listed in Table 1. These results indicate that the performance of Super P is superior to that of Vulcan XC500. In particular, the discharge capacity of Super P is almost twice that of Vulcan XC500. Super P, with a large specific surface area, could provide more reaction sites for Li^+^ ions from the anode (Wang et al., 2018). Subsequently, as shown in Figure 2B, L5SC was used as a catalyst and mixed with Vulcan XC500 and Super P, and the obtained cathode was tested. The results show that the addition of L5SC improved both the charge and discharge capacities. In the case of Vulcan XC500, the charge capacity increased from 926 to 2,247 mAh g^−1^, and the discharge capacity increased from 1,394 to 2,108 mAh g^−1^. This indicates that L5SC can promote the OER and ORR in Li−O_2_ batteries. Furthermore, in the case of Super P, the charge and discharge capacities were significantly enhanced from 955 to 5,500 mAh g^−1^ and from 2,253 to 6,032 mAh g^−1^, respectively. Super P has a large specific surface area (62 m^2^/g) for mixing with L5SC; therefore, the addition of L5SC produced a greater effect on OER and ORR of the Li−O_2_ battery, resulting in excellent Li−O_2_ battery performance.

**TABLE 1:** Capacities obtained in the charge–discharge test for carbon black (XC500 or Super P) air electrodes without and with L5SC.

Samples	Charge capacity (mA h g^–1^)	Discharge capacity (mA h g^–1^)
XC500	926	1,394
Super P	955	2,253
L5SC/XC500	2,247	2,108
L5SC/Super P	5,500	6,032

**FIGURE 3:**
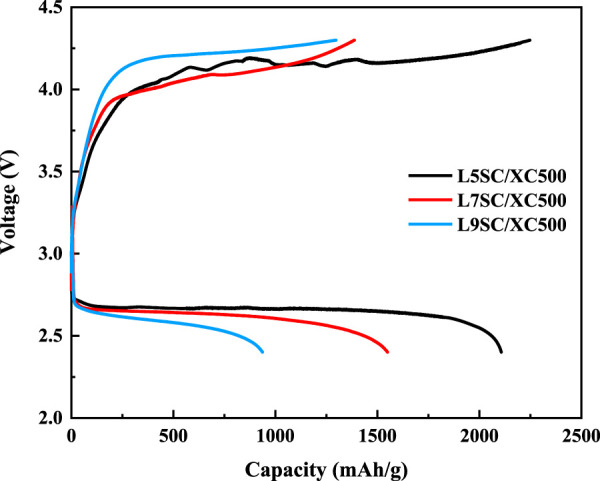
One-cycle charge–discharge test results for Vulcan XC500 cathode with different catalysts (L5SC, L7SC, and L9SC).

The capacities of cathodes made of Vulcan XC500 mixed with different catalysts (L5SC, L7SC, and L9SC) were measured using one-cycle charge–discharge tests (Figure 3; Table 2). According to the results, the capacities show the same tendencies during charge and discharge. The L5SC/XC500 electrode shows the best performance, and the L7SC/XC500 electrode shows a higher capacity than the L9SC/XC500 electrode. These results suggest that catalyst activity could be promoted by increasing the number of oxygen vacancies. It has been previously shown that the catalytic activity of Sr-doped LaCoO_3_ in the OER could increase with the number of oxygen vacancies (Mefford et al., 2016; Lu et al., 2019). Furthermore, the number of oxygen vacancies in perovskites has also been reported to be related to catalytic performance for ORR (Gayen et al., 2020; Ji et al., 2020). Therefore, it was considered that the charge and discharge capacities increased because the oxygen vacancies in Sr-doped LaCoO_3_ promoted the OER and ORR of the Li–O_2_ batteries.

**TABLE 2:** Charge–discharge capacity of the Vulcan XC500 cathode with different catalysts (L5SC, L7SC, and L9SC).

Samples	Charge capacity (mA h g^–1^)	Discharge capacity (mA h g^–1^)
L5SC/XC500	2,247	2,108
L7SC/XC500	1,384	1,550
L9SC/XC500	1,297	937

**FIGURE 4:**
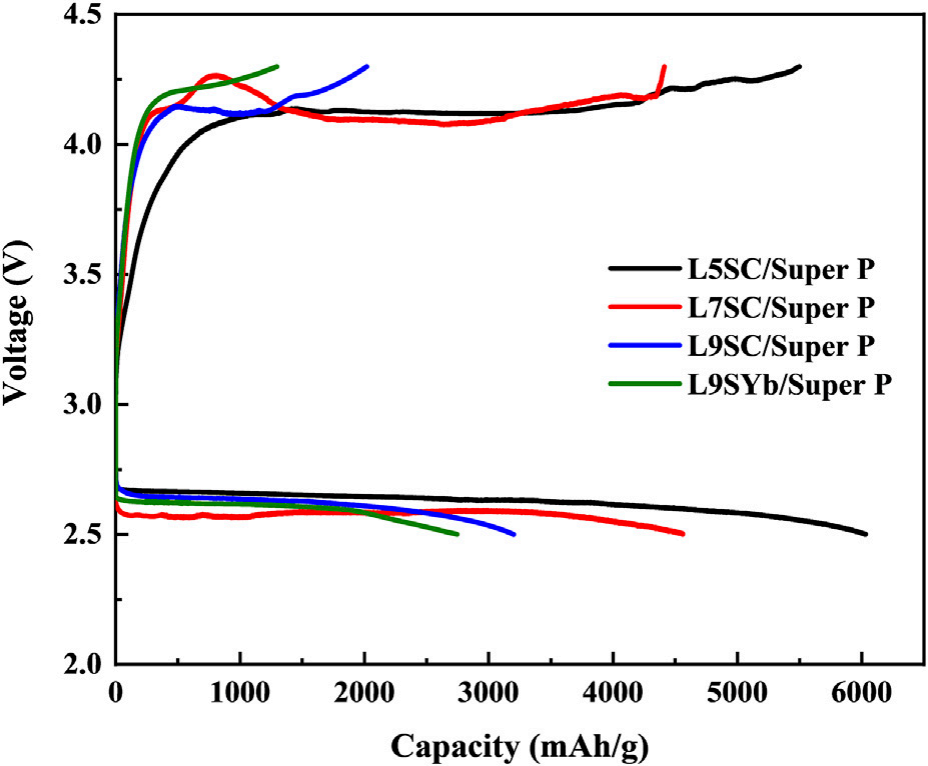
Capacity of Super P cathode with different catalysts (L5SC, L7SC, L9SC, and L9SYb) in one-cycle charge–discharge test.

The capacities of cathodes made of Super P mixed with different catalysts (L5SC, L7SC, L9SC, and L9SYb) were measured using one-cycle charge–discharge tests (Figure 4; Table 3). According to the results, the capacities show the same tendencies during charge and discharge. The L5SC/Super P demonstrated the highest capacity in this study. This is because L5SC has the highest catalytic activity in the OER and ORR, and Super P has a larger specific surface area than Vulcan XC500. In addition, compared with Sr-doped LaCoO_3_, L9SYb shows relatively low catalyst activity in OER and ORR. In fact, the OER and ORR performances of L9SYb were previously investigated in only a few studies because of its low catalytic activity. Therefore, the effects of the catalysts can be easily elucidated by comparing the differences between the performances of L5SC and L9Yb.

**TABLE 3:** Capacities of Super P cathodes with different catalysts (L5SC, L7SC, L9SC, and L9SYb) in charge–discharge tests.

Samples	Charge capacity (mA h g^–1^)	Discharge capacity (mA h g^–1^)
L7SC/Super P	4,413	4,562
L9SC/Super P	2019	3,202
L9SYb/Super P	1,297	2,746

**FIGURE 5:**
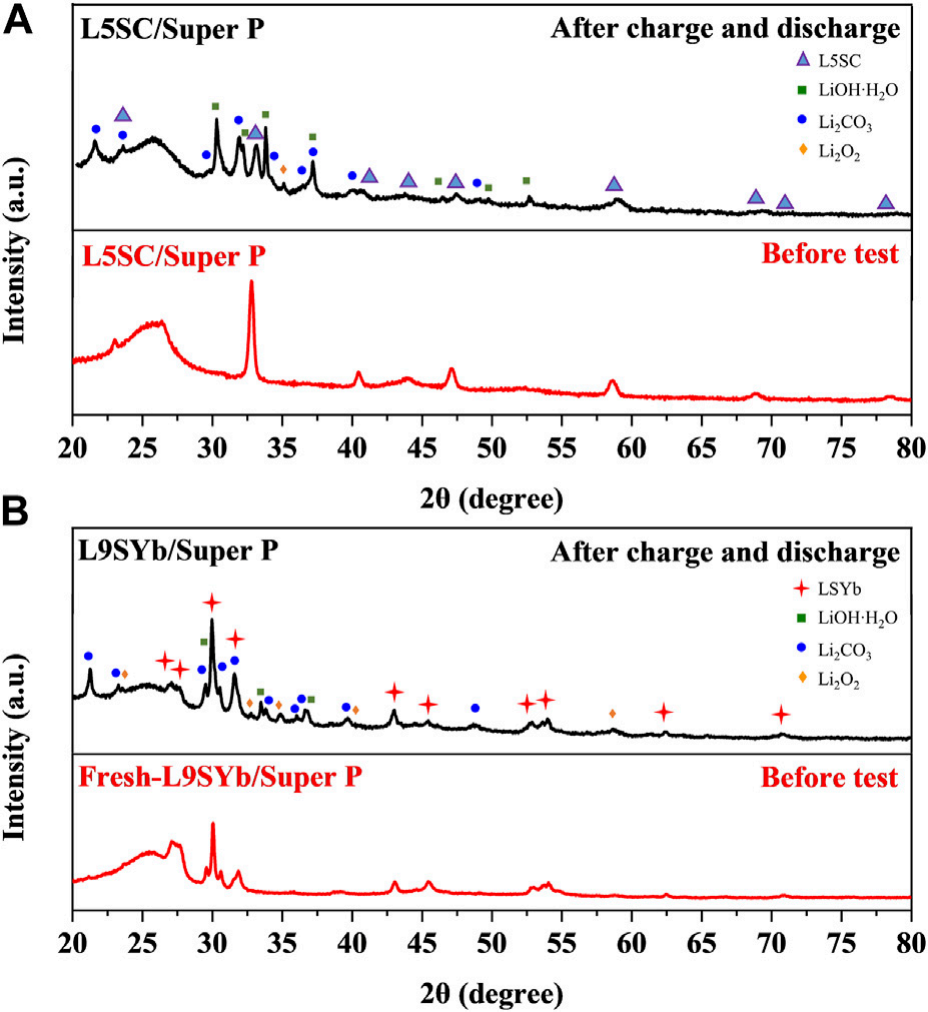
XRD results before and after one-cycle charge–discharge test for **(A)** L5SC/Super P and **(B)** L9SYb/Super P.

**FIGURE 6:**
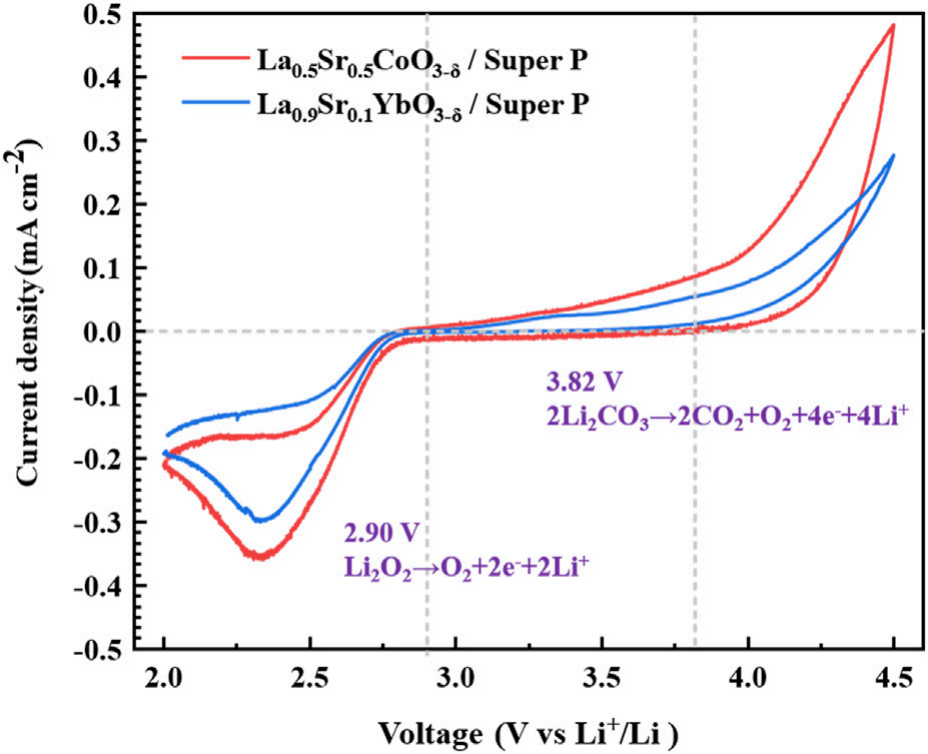
CV results for Li–O_2_ batteries with L5SC/Super P and L9SYb/Super P.

Figure 5 shows XRD results for L5SC/Super P and L9SYb/Super P before and after one-cycle charge–discharge test. In Figure 5, L5SC and L9SYb are labeled with blue triangle and red cross, respectively, and Li_2_CO_3_ and Li_2_O_2_ are labeled with blue circle and orange diamond, respectively. The comparison of Figures 5A, B shows that more Li_2_CO_3_ was generated on the L5SC/Super P cathode than on the L9SYb/Super P cathode. This indicates that L5SC promoted the reaction between C (from electrolyte solvent or cathode material), O_2_, and Li^+^ to generate Li_2_CO_3_ during discharge. According to the literature, the generation of Li_2_CO_3_ can increase the capacity of Li/CO_2_–O_2_ batteries (Takechi et al., 2011; Zou et al., 2019). Yin et al. suggested that two electrons are involved in the generation of Li_2_CO_3_ (Yin et al., 2017). At the same time, only one electron is involved in the formation of Li_2_O_2_. Therefore, the generation of Li_2_CO_3_ could significantly increase the discharge capacity of L5SC/Super P catalysts. We could not determine the amount of Li_2_CO_3_ produced through XRD measurements; therefore, we conducted CV experiments on Li–O_2_ batteries with L5SC/Super P and L9SYb/Super P cathodes (Figure 6).

CV curves for Li–O_2_ batteries with L5SC/Super P and L9SYb/Super P were obtained in the voltage range of 2.0–4.5 V at a constant current of 0.1 mA cm^−2^ (Figure 6). To observe complete reduction and oxidation peaks in CV curves, they were obtained in the potential range of 4.5–2.0 V. The ORR peak in CV curves corresponds to the generation of Li_2_O_2_ and Li_2_CO_3_, while the OER peak corresponds to the evolution of O_2_ and CO_2_ from Li_2_O_2_ and Li_2_CO_3_. In addition, according to previous studies, the theoretical voltage for Li_2_O_2_ oxidation is 2.90 V for the reaction Li_2_O_2_ → 2Li^+^ + 2e^−^ + O_2_ (Lim et al., 2013). At the same time, the theoretical voltage for the oxidation of Li_2_CO_3_ is 3.82 V through the reaction Li_2_CO_3_ → 2Li^+^ + 2e^−^ + 1/2O_2_ + CO_2_ (Lim et al., 2013). Therefore, Li_2_CO_3_ is more chemically stable than Li_2_O_2_. The mentioned voltage levels (2.9 and 3.82 V) are indicated in Figure 6. In Figure 6, for both cathodes, the current density during oxidation increased at 2.9 V and reached a maximum at 4.5 V. In particular, for L5SC/Super P, the current density significantly increases in the voltage range of 3.82–4.5 V. In addition, the CV curve area of the L5SC/Super P battery is larger than that of the L9SYb/Super P battery. This indicates that a greater amount of generators in the L5SC/Super P cathode was oxidized and decomposed to Li ions and gases during oxidation. Above 3.82 V this phenomenon is more pronounced. This suggests that more Li_2_CO_3_ was produced in the L5SC/Super P cathode. Therefore, the L5SC/Super P cathode demonstrated the best discharge capacity in this study because of promoted Li_2_CO_3_ generation.

**FIGURE 7:**
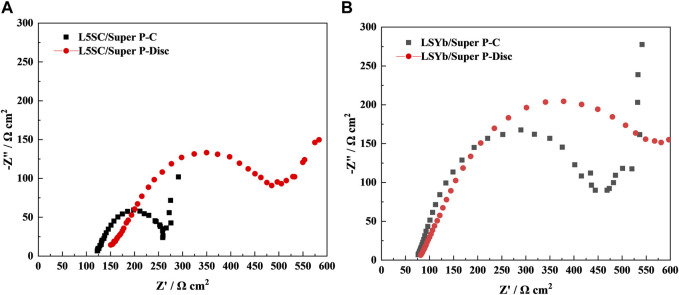
EIS results during charge and after discharge for **(A)** L5DC and **(B)** L9SYb.

The electrochemical impedance spectroscopy (EIS) results during charge and after discharge are shown in Figure 7. For L5SC/Super P, the ohmic and polarization resistances significantly increase after discharge. This indicates that a greater amount of non-conductive Li_2_O_2_ and Li_2_CO_3_ was generated in L5SC/Super P, which blocked electronic conduction and O_2_ diffusion. As shown in Figures 7A, B, the polarization resistance of L9SYb/Super P is larger than that of L5SC/Super P, which also indicates that L9SYb has lower catalytic activity than L5SC.

## 4 Conclusion

In this study, the charge–discharge performances of L9SC, L7SC, L5SC, and L9SYb catalysts for Li-O_2_ batteries were investigated. In addition, the charge–discharge performances of two types of carbon black (XC500 and Super P) were determined. According to the literature, La_1-x_Sr_x_CoO_3-δ_ is as a superior OER and ORR catalyst compared to La_0.9_Sr_0.1_YbO_3-δ_. Additionally, an increase in the number of oxygen vacancies and an increase in the surface area by blending with Super P carbon have been previously reported to improve the catalytic activity in cathodic reactions in Li–O_2_ batteries. In this study, the L5SC/Super P air electrode showed the best charge and discharge capacities corroborating the abovementioned findings from the literature. In addition, the type of generator (Li_2_O_2_ or Li_2_CO_3_) could be considered a factor that affects the discharge capacity. The results of this study show that the battery capacity increases with the amount of Li_2_CO_3_ generated. The L5SC/Super P cathode material promoted the production of Li_2_CO_3_ and thus showed excellent performance. At the same time, based on XRD and CV results, L9SYb/Super P showed low Li_2_CO_3_ yields, which also indicated that it did not promote the reaction of Li^+^ ions with oxygen.

## Data Availability

The raw data supporting the conclusion of this article will be made available by the authors, without undue reservation.
